# Monitoring the Velocity of Domain Wall Motion in Magnetic Microwires

**DOI:** 10.3390/s24041326

**Published:** 2024-02-19

**Authors:** Alexander Chizhik, Paula Corte-Leon, Valentina Zhukova, Juan Mari Blanco, Arcady Zhukov

**Affiliations:** 1 Department Advanced Polymers and Materials: Physics, Chemistry and Technology, University of Basque Country UPV/EHU, 20018 San Sebastian, Spain; paula.corte@ehu.eus (P.C.-L.); valentina.zhukova@ehu.eus (V.Z.); arkadi.joukov@ehu.eus (A.Z.); 2 Department of Applied Physics, University of Basque Country EIG, UPV/EHU, 20018 San Sebastian, Spain; juanmaria.blanco@ehu.eus; 3 IKERBASQUE, Basque Foundation for Science, 48011 Bilbao, Spain

**Keywords:** soft magnetic materials, amorphous magnetic microwires, magnetic domains, magneto-optic Kerr effect, magnetic anisotropy

## Abstract

An approach was proposed to control the displacement of domain walls in magnetic microwires, which are employed in magnetic sensors. The velocity of the domain wall can be altered by the interaction of two magnetic microwires of distinct types. Thorough investigations were conducted utilizing fluxmetric, Sixtus–Tonks, and magneto-optical techniques. The magneto-optical examinations revealed transformation in the surface structure of the domain wall and facilitated the determination of the mechanism of external influence on the movement of domain walls in magnetic microwires.

## 1. Introduction

Amorphous soft magnetic materials, such as glass-coated microwires and ribbons, play a fundamental role in numerous technological applications [1,2,3,4,5,6,7]. The most advanced application of these soft magnetic materials is their utilization in magnetometers and magnetic sensors [8,9,10,11,12,13]. This utilization is made possible by the exceptional magnetic properties and good mechanical properties of the materials, as well as the existence of a well-established and validated production and quality control system.

The perfectly cylindrical shape of magnetic wires presents the opportunity to observe magnetic properties that are quite unusual, such as spontaneous magnetic bistability and/or the giant magnetoimpedance (GMI) effect [1,14,15,16,17,18,19]. These properties are intrinsically linked to the distinctive domain structure of magnetic wires, which consists of an inner axially magnetized core surrounded by an outer domain shell [14,20]. Consequently, the high GMI effect of Co-rich magnetic wires is a result of the high circumferential magnetic permeability of Co-rich amorphous wires [14,20,21,22,23,24]. On the other hand, spontaneous magnetic bistability is attributed to the remagnetization process within the axially magnetized core brought about by the rapid propagation of domain walls (DWs) [1,20].

The observation of rapid single-domain wall (DW) propagation in magnetic wires has garnered significant attention from the perspective of fundamental physics. This includes investigating the origins of DW nucleation and propagation fields, as well as the remarkably high DW velocities (*v*) and DW mobility (*S*) [1,20,25,26,27,28,29,30]. Conversely, various potential applications, such as racetrack memories, magnetic logic, and electronic surveillance, have been developed by leveraging the magnetic bistability of fast and controllable DW propagation [31,32,33].

In the majority of applications, the efficient regulation of individual domain walls (DWs) through injection, the management of controllable DW propagation, and the act of pinning are of utmost significance [31,32,34].

Generally, this study aligns with the overarching concept of interlayer interaction present in magnetic multilayer structures featuring non-magnetic separators surpassing exchange lengths [35,36]. Notably, oscillation periods of interlayer exchange were noted, contingent upon the non-magnetic material type and thickness. In our investigation, the glass coating of two microwires functioned as the non-magnetic layer. Additionally, within this framework lies the notion of employing two magnetic microwires with distinct chemical compositions, hence differing magnetic characteristics.

One of the primary focal points in our previous research, within the context of the specified orientations of employing magnetic microwires, entailed a comprehensive examination of the dynamics of domain walls (DWs), both internally within the microwires and on their surfaces [34,37]. Particularly noteworthy to us was the matter of regulating DW propagation [31,34,37,38,39]. In the course of developing diverse methodologies for such regulation, we pursued the concept of reciprocal influence between closely positioned microwires. This concept was explored not only through our own research endeavors [37], but also by other scientific communities [40,41,42], thereby enabling us to assess the potential level of this reciprocal influence in relation to the distance separating the microwires.

In the majority of prior publications, researchers have investigated the magnetostatic interaction among microwires that possess similar compositions and geometry [37,40,41,42,43,44]. In terms of the dynamics of domain walls, our previous work from several years ago demonstrated that the relationship between the velocity of the DW and the applied magnetic field could be influenced by the magnetostatic interaction with another microwire with identical properties [37]. Furthermore, only a small number of publications have reported on the distinctive magnetic properties observed in a linear array consisting of microwires with varying chemical compositions and magnetic properties [45].

As an outcome, the notion of situating two microwires with distinct characteristics in close proximity to each other appeared innovative and promising to us. In order to achieve a located influence, one of the microwires was deliberately chosen to be considerably shorter than the other. Consequently, one of the designated wires was a lengthy Fe-rich microwire. In this microwire, which possesses the magnetic bistability phenomenon, we examined the displacement of an individual DW, the presence of which we were already aware of. The second microwire, with which we aimed to exert a localized influence, was a short Co-rich microwire.

We opted for a specific set of research techniques that appeared most appropriate for this study, including the fluxmetric method [46], the Sixtus–Tonks method [37], and the magneto-optical Kerr effect technique [47].

## 2. Experimental Details

**Figure 1:**
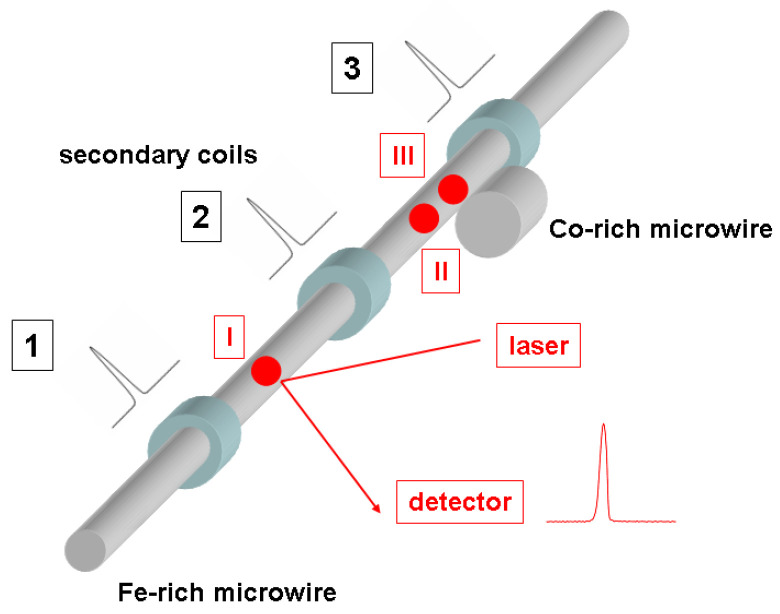
Schematic picture of the experimental set-up: 1, 2, and 3—pick-up coils; I, II, and III, points of laser reflection during the MOKE experiment. The position of the Co-rich microwire relative to the pick-up coils and the Fe-rich microwire is demonstrated.

In our studies, we used the following two microwires: an as-prepared 12 cm long Fe_75_B_9_Si_12_C_4_ microwire (diameter of the metallic nucleus *d* = 19.8 µm, total diameter *D* = 28.6 µm) and a 1 cm long Co_64.6_Fe_5.8_B_16.8_Si_11_Cr_3.4_ microwire (*d* = 80 µm, *D* = 92.3 µm). The Co-rich microwire was located in close proximity to the Fe-rich microwire (Figure 1). 

The magnetic hysteresis loops were obtained utilizing the fluxmetric technique, which has been previously employed for the characterization of magnetically soft microwires. The investigation of the magnetization reversal process in the surface region of the microwire was conducted using a MOKE loop tracer. The polarized light emitted by a He–Ne laser was directed towards the detector after being reflected from the microwire (Figure 1).

The DW velocity was determined using a modified Sixtus–Tonks experiment, which has previously proven effective in the examination of DW dynamics in magnetic microwires [20,26]. The microwire was positioned within an extended solenoid, establishing a magnetic field.

Three pickup coils were positioned coaxially within the solenoid (Figure 1), encircling the sample and kept at an equal distance apart, for the purpose of evaluating the velocity of the DW. The velocity of the DW was determined by observing the difference in time between the electromotive force (*EMF*) peaks caused in the pick-up coils by the displacement of the DW. The pick-up coils were separated by the distance *L*.

In addition to the phenomenon of surface hysteresis, the MOKE method facilitates the detection of MOKE peaks that correspond to the displacement of a domain wall across the surface of the specimen. By acquiring knowledge about the characteristics of the MOKE peaks at various positions on the surface of the elongated microwire sample (designated as locations I, II, and III in Figure 1), we employed the MOKE technique to infer the structure of the domain wall at the different aforementioned locations within the investigated microwire.

## 3. Results and Discussion

**Figure 2:**
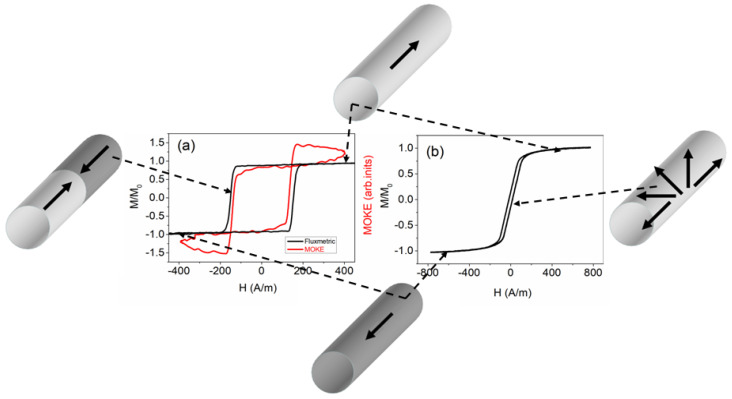
Fluxmetric (black lines) and MOKE (red line) hysteresis loops obtained from Fe-rich (**a**) and Co-rich (**b**) samples.

As an initial investigation, magnetic and magneto-optical hysteresis were acquired from the two samples utilized in the experiments. Figure 2 shows the hysteresis loops for the magnetic (represented by black lines) and MOKE (represented by red line) methods. The Fe-rich microwire, which displayed positive magnetostriction, exhibited a volume hysteresis loop with a perfectly rectangular shape (Figure 2a, black line). Conversely, the Co-rich microwire, with a vanishing value of magnetostriction, exhibited an inclined hysteresis loop with significantly lower coercivity (Figure 2b). The shape of the volume hysteresis loop observed for the Fe-rich microwire is associated with the so-called “magnetic bistability effect”.

Figure 2 depicts diagrams illustrating the domain structures. The black arrows represent magnetization in a schematic manner. When the external magnetic field reached a sufficient magnitude, both types of microwires reached saturation (top and bottom inserts). This is denoted by the black arrows that align with the microwire axis. In Fe-rich microwires, magnetization reversal occurs through the swift movement of a flat domain wall separating domains with opposite magnetization (as shown in the left inset). Generally, in Co-rich microwires, various domain structures, such as axial, helical, or circular structures, may exist. However, in this instance, magnetization reversal occurs through the rotation of magnetization without forming a stable domain structure (as shown in the right inset).

The process of magnetization reversal in Fe-rich microwires takes place when a single DW is detached from one of the closure end domains and is subsequently displaced along the microwire [20,26,48,49]. On the other hand, the magnetization reversal process in Co-rich microwires is characterized by the rotation of magnetization. As a result, these microwires typically exhibit low coercivity and high magnetic permeability [50,51,52,53].

It was equally important to obtain pertinent details regarding the process of magnetization reversal within the surface layer of a Fe-rich microwire. The confirmation of our assumptions regarding the magnetic bistability, referred to as the “surface bistability effect”, was made evident by the rectangular shape observed in the MOKE hysteresis loop. This observation was made both in the sample’s volume and on its surface. Therefore, we deduced that during the magnetization reversal, there was swift displacement of a solitary domain wall on the surface of the Fe-rich sample, as well as within the bulk. It is important to highlight the slight deviation of the signal from a linear path, as observed in the MOKE hysteresis loop. This occurred because the laser beam reflected not from a flat surface, but from a cylindrical surface of the sample. Nonetheless, this did not hinder our ability to detect magnetization jumps on the sample’s surface that were linked to the rapid movement of the domain wall. The slight disparity in the coercivity magnitude observed in the magnetic and MOKE hysteresis loops arose from the fact that the process of magnetization reversal initiates from the surface of the Fe-rich sample.

**Figure 3:**
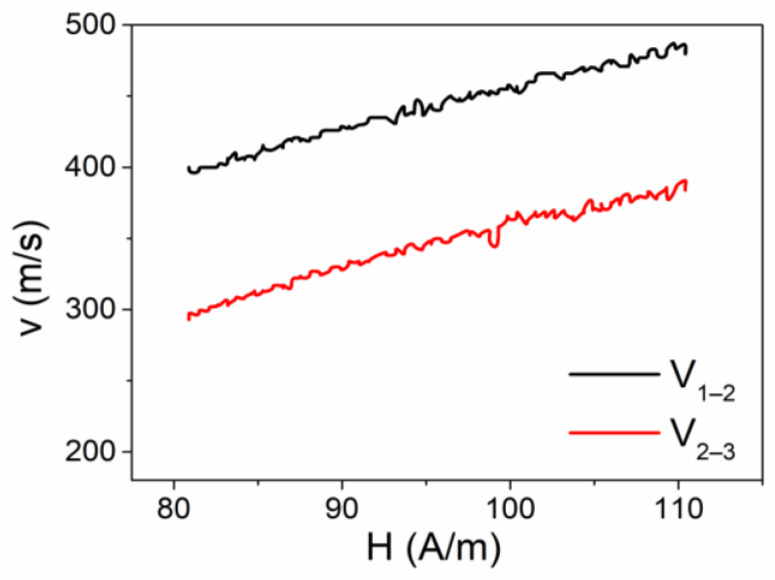
V(H) dependencies obtained from the Fe-rich microwire. V_1–2_ represents the velocity dependence obtained between secondary coils 1 and 2. V_2–3_ represents the velocity dependence obtained between secondary coils 2 and 3.

After obtaining initial insights into the bulk and surface magnetization reversal process, we initiated an investigation into the propagation of the domain wall (DW) using the Sixtus–Tonks method. In Figure 3, the magnetic field dependencies of velocity in the Fe-rich sample are illustrated. The placement of the Co-rich short wire aligns with the diagram depicted in Figure 1.

The Co-rich microwire was situated in close proximity to the surface of the long Fe-rich microwire, as well as in the space between the secondary coils 2 and 3. The black line (Figure 3) represents the velocity that was measured between coils 1 and 2, whereas the red line (Figure 3) was measured in the region of the sample between coils 2 and 3, where the shorter Co-rich microwire was situated. It can be observed that a substantial disparity in the value of the velocity of the domain wall, as measured in various regions of the sample, exists. In simpler terms, a controlled deceleration in the motion of the domain wall is apparent.

To examine the operation of regulated alterations in the speed of the domain wall, we investigated the conversion of the MOKE peaks acquired at different positions on the surface of the microwire.

**Figure 4:**
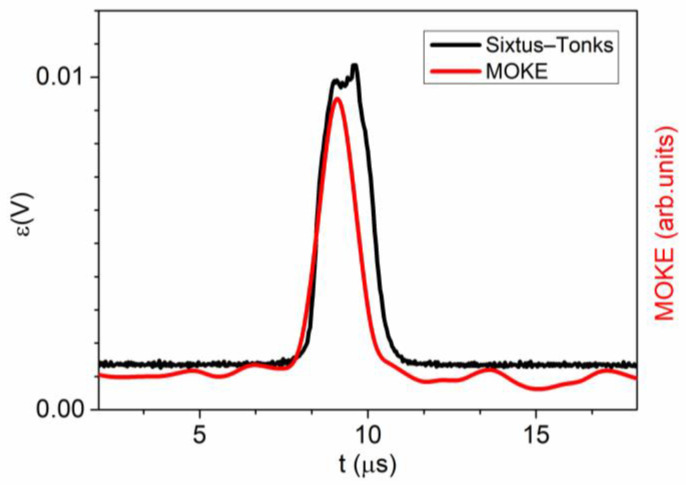
V(H) dependencies obtained in a linear array consisting of long Fe-rich and short Co-rich microwires. V_1–2_ represents the velocity dependence obtained between secondary coils 1 and 2. V_2–3_ represents the velocity dependence obtained between secondary coils 2 and 3.

The initial MOKE measurement (designated as point I in Figure 1) was carried out between coils 1 and 2. A comparison of the EMF peak registered by coil 1 and the MOKE peak registered at point I is shown in Figure 4. It is evident that the shapes of these two signals bear significant resemblance. This observation suggests the rapid, homogeneous, and compact motion of the domain wall within the volume of the microwire and on its surface. In this particular instance, the MOKE peak exhibited a somewhat narrower width compared to the peak acquired using the Sixtus–Tonks technique. Presumably, this discrepancy arose from the fact that the width of the domain wall on the sample’s surface was slightly smaller than within its interior.

In the second phase of the MOKE investigation, our focus shifted towards the region between coils 2 and 3, which exhibited a lower speed of the domain wall. The MOKE peaks were obtained at the designated locations II and III, as illustrated in Figure 1. Point II can be identified on the surface of the Fe-rich microwire, positioned in close proximity to the Co-rich microwire. On the other hand, point III was situated on the surface of the Fe-rich microwire, slightly above the Co-rich microwire. Our belief is that this particular area of the Fe-rich microwire, encompassing points II and III, found itself within the zone of influence exerted by the Co-rich microwire, albeit to a varying degree at each point.

**Figure 5:**
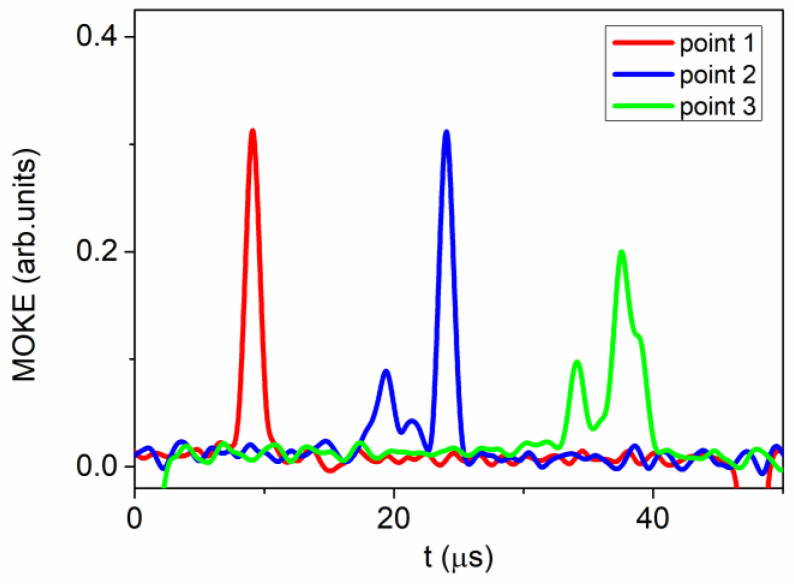
V(H) dependencies obtained in a linear array consisting of long Fe-rich and short Co-rich microwires. V_1–2_ represents the velocity dependence obtained between secondary coils 1 and 2. V_2–3_ represents the velocity dependence obtained between secondary coils 2 and 3.

The findings of these MOKE experiments are depicted in Figure 5. It is evident that the morphology of the MOKE peaks exhibits variability contingent upon the precise location of the observation point. As previously mentioned, a consistent peak achieved at point I corresponds to the displacement of the compact and regular domain wall. Conversely, the signal received at point II (the blue line in Figure 5) exhibits a modified shape in relation to the peak obtained at point I. An additional series of smaller peaks and a reduction in the amplitude of the primary peak signified a metamorphosis of the domain wall on the surface of the Fe-rich microwire. The signal received at point III (the green line in Figure 5) underwent even more pronounced alterations. At this juncture, a series of peaks with varying heights became apparent. Simultaneously, the primary peak continued to decrease.

**Figure 6:**
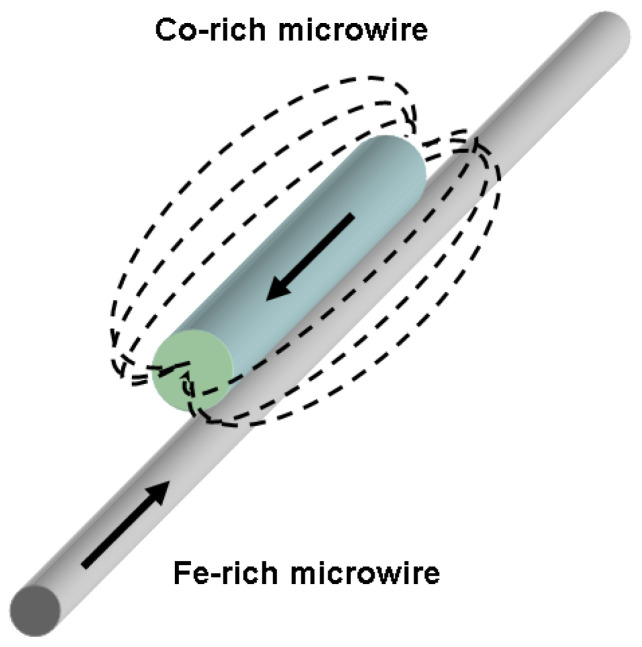
Schematic sketch of stray field distribution.

Henceforth, the alterations observed in the MOKE signal imply a metamorphosis of the surface segment of the domain wall within the sphere of impact of the Co-rich microwire. Evidently, the impact of the Co-rich microwire is associated with its stray fields (Figure 6); however, certain peculiarities exist.

We maintain the belief that the impact of stray fields is non-uniform, both in terms of the microwire’s length and diameter. In the vicinity of the Co-rich microwire (designated as point II in our experimental setup), the configuration of domains was primarily influenced by the stray magnetic field that was perpendicular to the microwire’s axis. As we traversed the surface of the microwire (designated as point III in our experiment), the contribution of the longitudinal stray magnetic field became increasingly prominent. In this particular scenario, the surface region of the microwire, which we examined using the MOKE method, was situated within a region exhibiting significant variations in the amplitude of the magnetic field.

The observed transformation of the domain wall manifested through the emergence of a specific form of non-uniformity. In the region where the perpendicular stray fields exerted their effect (identified as point II), local areas with a perpendicular component of magnetization appeared on the domain wall. Based on the minor peak count, there were two such regions. At point III, two minor peaks transitioned into a singular peak; however, simultaneously, the primary peak widened and diminished in magnitude, thereby indicating an augmentation in the influence of the stray field that aligned parallel to the sample’s axis.

Generally speaking, we posit that the primary impact of a closely positioned additional microwire lies in the induced complexity of the moving domain wall’s shape. The key elements of this mechanism involve the creation and enlargement of regions with uneven magnetization rotation within the domain wall, together with its general broadening.

## 4. Conclusions

We proposed a novel method for controlling the movement of domain boundaries in magnetic microwires. Our investigation focused on understanding the impact of the Co-rich microwire’s proximity on the velocity of the domain wall in the Fe-rich microwire. Various experimental techniques, including the fluxmetric method, Sixtus–Tonks method, and magneto-optical Kerr effect method, were employed. These complementary methods allowed us to ascertain the key properties of the influence of the Co-rich microwire and, more importantly, comprehend the underlying mechanism. Our findings revealed a noticeable alteration in the domain wall velocity within the region directly adjacent to the additional Co-rich microwire in the studied sample.

During these experiments, we observed a notable alteration in the magneto-optical peaks linked to the motion of the domain wall in the surface region of the microwire. We interpreted this change in MOKE peaks as a complexity emerging in the structure of the surface segment of the domain wall as it neared the influence zone of the additional microwire.

Ultimately, alterations in the moving domain wall’s structure resulted from the combination of longitudinal and transverse projections of the stray magnetic field, enabling local control over its velocity. We posit that when applied to a system comprising numerous microwires with diverse properties, this method holds promising potential.

## Data Availability

Data is available upon request due to restrictions related to development.
